# Differences in Upgrading of Prostate Cancer in Prostatectomies between Community and Academic Practices

**DOI:** 10.1155/2013/471234

**Published:** 2013-10-24

**Authors:** Franklin Lee, Henry Gottsch, William J. Ellis, Lawrence D. True, Daniel W. Lin, Jonathan L. Wright

**Affiliations:** ^1^Department of Urology, University of Washington School of Medicine, Health Sciences Building, 1959 NE Pacific, BB-1115, P.O. Box 356510, Seattle, WA 98195, USA; ^2^Department of Pathology, University of Washington School of Medicine, Seattle, WA 98195, USA; ^3^Public Health Sciences, Fred Hutchinson Cancer Research Center, Seattle, WA 98109, USA

## Abstract

*Objective*. To determine whether initial biopsy performed by community or academic urologists affected rates of Gleason upgrading at a tertiary referral center. Gleason upgrading from biopsy to radical prostatectomy (RP) is an important event as treatment decisions are made based on the biopsy score. *Materials and Methods*. We identified men undergoing RP for Gleason 3 + 3 or 3 + 4 disease at a tertiary care academic center. Biopsy performed in the community was centrally reviewed at the academic center. Multivariate logistic regression was used to determine factors associated with Gleason upgrading. *Results*. We reviewed 1,348 men. There was no difference in upgrading whether the biopsy was performed at academic or community sites (OR 0.9, 95% CI 0.7–1.2). Increased risk of upgrading was seen in those with >1 positive core, older men, and those with higher PSAs. Secondary pattern 4 and larger prostate size were associated with a reduction in risk of upgrading. Compared to the smallest quartile of prostate size (<35 g), those in the highest quartile (>56 g) had a 49% reduction in risk of upgrading (OR 0.51, 95% CI 0.3–0.7). *Conclusion*. There was no difference in upgrading between where the biopsy was performed and community and academic urologists.

## 1. Introduction

Prostate cancer risk stratification prior to definitive treatment is crucial as treatment selection relies on these factors. Whereas the PSA and clinical stage can be easily repeated with minimal patient risk, repeating the prostate needle biopsy to confirm accurate Gleason grading is much more invasive. Thus, adequate sampling of the prostate during biopsy is paramount.

Despite improvements in sampling techniques at prostate needle biopsy, discordance between the diagnostic biopsy Gleason score and radical prostatectomy (RP) Gleason score occurs in up to 40% of the cases [1]. Several factors are associated with an increased risk of pathologic upgrading. These include smaller prostates [2, 3], higher PSAs [3, 4], and higher volume cancer at biopsy [5]. In addition, interobserver variability in pathologic interpretation of PCa specimens plays a role in this discordance [6–8]. Central pathologic review by dedicated genitourinary pathologists has been shown to lead to more accurate grading of the biopsy Gleason score and subsequent higher concordance with RP Gleason score [9]. It is now common for tertiary centers to require internal review of all outside biopsies prior to treatment. 

Several technical aspects to improve prostate sampling have been instituted including laterally directed biopsies [10], increased number of biopsy cores taken [11–13], anterior apical biopsies [14, 15], and transition zone biopsies in selected cases [16]. To what extent these techniques have been adopted by both academic and community urologists is unknown. If there existed a higher risk of upgrading at RP in cases when the biopsy was not performed at a high volume academic PCa center, this could result in a consideration for repeat biopsies prior to RP for cases referred to such tertiary centers. To evaluate this source of variance, we compare Gleason upgrading from biopsy to RP with specific focus on whether the biopsy was performed at a high volume PCa academic center or in the community. 

## 2. Methods

### 2.1. Study Population

The cohort was identified from an institutional database of men undergoing RP for PCa after obtaining IRB approval for the study. All men had both biopsy and RP pathology reviewed at the central institution. Those who had biopsies performed by community providers had central review and internal Gleason score recorded. The analysis was limited to those with biopsy Gleason scores of 3 + 3 and 3 + 4. 

Patients were considered to have experienced upgrading at RP if they had an increase in Gleason sum of 1 point or greater or if Gleason score increased from 3 + 4 to 4 + 3. In addition, men who had tertiary pattern 5 (TP5) at RP were considered to have been upgraded. We recorded the number of biopsies taken, the number of biopsy cores positive for PCa, and the year of biopsy. Diagnostic PSA and prostate volume on transrectal ultrasound were collected. PSA was categorized as <4.0, 4.0–9.9, and ≥10. Prostate volume was categorized by quartile and age, grouped into 10- year increments (<60, 60–69, ≥70). 

### 2.2. Statistical Analysis

Differences in the clinical and pathologic factors were determined using the chi-square test by site (academic or community) of prostate needle biopsy. Multivariate, logistic regression was performed to determine which factors were associated with pathologic upgrading at RP. Missing data was present in less than 3% of the variables age, number of positive cores, prostate volume, and PSA. A higher percentage of data was missing from the number of positive cores (18% of both those experiencing upgrading and those not experiencing upgrading). Because of this higher number, a dummy variable was created to account for missing data in the multivariate model. All statistical analyses were conducted using Stata software version 11.0.

## 3. Results

In total, there were 1,348 patients that underwent RP at our academic center for Gleason 3 + 3 (*n* = 905, 67%) or 3 + 4 (*n* = 443, 33%) PCa. The majority of men had their prostate biopsy in the community (63%) rather than the academic center (37%). Table 1 shows the distribution of clinical and biopsy characteristics between those biopsied in the community practice versus those at the academic site. Those undergoing biopsy in the community were more commonly younger, with smaller prostates, and lower PSA values (all *P* < 0.001).

Overall, the proportion of men experiencing upgrading was 34%. The majority of those with upgrading (74%) had Gleason 3 + 3 on biopsy. Of those upgraded from Gleason 3 + 3, the majority (79%) increased to Gleason 3 + 4, whereas upgrading to 4 + 3 or 8–10/tertiary pattern 5 accounted for only 9% and 12% of all those upgraded from Gleason 3 + 3, respectively. For those with Gleason 3 + 4 disease at biopsy who were upgraded (*n* = 118), 47% were upgraded to Gleason 4 + 3 and 53% to Gleason 8–10/TP5. A total of 82 men had TP5 present in the RP specimen. However, in only 37 men (2.7% of entire cohort) was the TP5 responsible for categorization as upgrading. The remaining 45 men with TP5 present had upgrading criteria met by the primary or secondary Gleason grade change.

Table 2 shows the pathologic and clinical characteristics of cases that had upgrading. Older age, higher PSAs, biopsy Gleason score 3 + 3, and having more than one biopsy core with cancer were all significantly associated with upgrading. The proportion of upgrading was similar between community and academic biopsy sites (32% and 35%, resp.). The results from the multivariate model are shown in Table 3. We found no difference in upgrading when comparing biopsies performed at the academic center versus community practices (OR 0.91, 95% CI 0.7–1.2). the number of biopsies obtained was not associated with upgrading (OR 0.8, 95% CI 0.6–1.2). Several variables were associated with Gleason upgrading at RP. Older men and those with higher PSAs had a greater risk of upgrading. Compared to men <60 years of age, those in their 60s (OR 1.4, 95% CI 1.1–1.8) and those older than 70 (OR 2.8, 95% CI 1.9–4.3) had an increasing risk of upgrading at RP. Similarly, compared to those with a PSA <4.0, a PSA of 4.0–9.9 (1.8, 95% CI 1.3–2.5) or ≥10 (OR 4.1, 95% CI 2.6–6.4) was associated with a greater risk of upgrading (pTrend < 0.001). Larger prostates and a secondary biopsy Gleason pattern 4 were both associated with a reduction in risk of upgrading at RP. Those in the highest quartile of prostate size (>56 cc) had a 49% reduction in risk of upgrading (OR 0.51, 95% CI 0.3–0.7) compared to those in the lowest quartile of prostate size (<35 cc).

Several subanalyses were performed to evaluate whether the lack of a relationship between site of biopsy and risk of upgrading persisted for different categories of patients (data not shown). These included limiting the analysis only to those with Gleason 3 + 3, excluding TP5 as a progression criteria, and limiting the analysis to those done in later years and to those with >10 biopsy cores taken. No differences in risk estimates were seen with these analyses.

## 4. Discussion

In this study, we found no difference in Gleason upgrading from prostate biopsy to RP between academic and community urologists. Considering the increasing literature on regionalization of urologic care, these data suggest that the dissemination of improved techniques for better sampling of the prostate during prostate needle biopsy to urologists has occurred both within and outside of high volume PCa centers. 

Gleason upgrading is well characterized and studies have identified several patient factors that are associated with upgrading including older age [1, 17], higher PSA [1, 17, 18] and larger prostates [1, 18, 19]. In our analysis, we also found these factors to be associated with the risk of upgrading. Compared to those with a PSA < 4.0, having a PSA > 10 was associated with a fourfold risk of upgrading (OR 4.1, 95% CI 2.6–6.4). Similarly, age was associated with upgrading. The oldest patients (>70) have a nearly threefold increase in risk of upgrading (OR 2.8, 95% CI 1.9–4.2) compared to those aged <60 years. Larger prostates were found to have a decreased risk of upgrading with the highest quartile of prostate size (>56 gm) found to have a 49% reduction in risk of upgrading (OR 0.51, 95% CI 0.3–0.7) compared to those in the lowest quartile of prostate size. The risk of upgrading has also been associated with pathologic findings including number of cores involved [20, 21], percentage of cores involved [20, 21], and the secondary Gleason pattern [1, 20, 21]. Our study demonstrated that the total number of cores involved was significantly associated with risk of upgrading. In addition, those who had secondary Gleason scores of 4 were found to have a decreased risk of upgrading, with a greater than 50% reduction in risk of upgrading (OR 0.39, 95% CI 0.3–0.52) compared to those with secondary Gleason pattern 3 disease. We did not have the percentage of cores involved on all cases and thus were not able to analyze this variable.

An important factor minimizing upgrading at tertiary centers has been employing central review of all biopsy specimens [9, 22]. Studies have demonstrated that centralized review of pathology specimens, with trained uropathologists, have decreased inter and intraobserver variability and improved concordance rates [9, 22]. Kuroiwa and colleagues collected data from 1629 patients who underwent radical prostatectomy. Biopsy and RP specimens were retrospectively reviewed by uropathologists. Concordance rates of centrally reviewed pathologic data were then compared to concordance rates obtained from local pathologists. Centrally reviewed pathologic data was found to have significantly improved concordance rates (65% versus 53% for Gleason 3 + 4 PCa). At our center, central review is required prior to RP and is recorded in the medical record, thus minimizing this variability. 

With increasing regionalization of PCa care, it is becoming more common for a large proportion of RPs performed at centers of excellence to be done on men whose biopsy was performed by a referring provider in the community. Decisions on choice of treatment, nerve sparing, and lymph node dissection are made based on the clinical prostate exam, PSA, and biopsy results. While PSAs and prostate exams can be readily confirmed by the consulting provider, adequacy of prostate gland sampling is unknown. Over the years, there have been several modifications to the standard biopsy technique described by Hodge et al in 1989 of systematic sampling from the base, mid, and apex along the mid parasagittal plane [23]. Several important modifications include increasing the number of cores, more laterally directed cores, and obtaining anterior apical cores [10–15]. Data regarding a specific biopsy template used within community practices were not available for this study. Overall, we found no difference in Gleason upgrading from community and academic urologists' prostate needle biopsy when the RP is performed at the high volume center. These data suggest that biopsy sampling and techniques are similar between academic and community sites of practice. 

This study has several limitations. It is a retrospective study and is limited to one institution. We did not have complete data on a number of variables including prostate volume, PSA, age, and number of positive cores. The percentage of cores involved or length of each core has been reported to be associated with risk of Gleason upgrading, and data regarding these variables would have also been ideal. Further, although we had central review of all pathology, we had several different academic pathologists interpreting the slides. Although this could introduce a source of error due to interobserver variability, we believe it reflects the current scenario that most high volume centers experience presently. Further, including a variable for our dedicated prostate pathologists in the model did not significantly change the risk estimates (data not shown). Finally, we do not have data on whether an end-fire or side-fire probe was used for the biopsies, which has been suggested to have an impact on the detection rate in recent publications [24].

## 5. Conclusion

In conclusion, while there are several factors associated with Gleason upgrading at time of RP, whether the biopsy was performed by an academic or community urologist was not associated with the risk of upgrading. These data suggest widespread adoption of techniques to improve adequate prostate sampling among urologists.

## Figures and Tables

**Table 1:** Distribution of clinical and biopsy characteristics between community and academic urologists.

	Academic *N* (%)	Community *N* (%)	*P* value*
Site of biopsy	502 (100)	846 (100)	
Age at biopsy			<0.01
<60	198 (39)	422 (49)	
60–69	220 (44)	350 (41)	
≥70	83 (17)	74 (9)	
PSA			<0.01
<40	92 (18)	182 (21)	
4–9.9	290 (58)	537 (63)	
≥10.0	112 (22)	95 (11)	
Prostate size (cc)			<0.01
<35	98 (20)	215 (25)	
35–44	121 (24)	235 (28)	
44–56	116 (23)	209 (25)	
>56	139 (28)	179 (21)	
Number of cores obtained			<0.01
≤6	252 (50)	276 (32)	
6–9	49 (10)	99 (12)	
10+	201 (40)	471 (56)	
Number of positive cores			<0.01
One	157 (31)	158 (19)	
Two	119 (24)	140 (17)	
Three	89 (18)	116 (14)	
Four or more	107 (12)	219 (26)	
Biopsy Gleason sum			<0.01
3 + 3	356 (71)	549 (65)	
3 + 4	146 (29)	297 (35)	

*P-value from chi-square test.

**Table 2:** Proportion of cases with Gleason upgrading at RP based on pathologic and clinical characteristics.

	Upgrading		*P*-value*
	No (%)	Yes (%)	
Age at biopsy			**<0.01**
<60	436 (49)	184 (41)	
60–69	371 (41)	199 (44)	
≥70	88 (10)	69 (15)	
PSA			**<0.01**
<4.0	208 (23)	66 (15)	
4–9.9	542 (61)	285 (63)	
≥10.0	114 (13)	93 (21)	
Prostate size (cc)			0.35
<35	196 (22)	117 (26)	
35–44	234 (26)	122 (27)	
44–56	218 (24)	107 (24)	
>56	220 (25)	98 (22)	
Number of cores obtained			**0.02**
≤6	373 (41)	155 (34)	
6–9	98 (11)	50 (11)	
10+	424 (47)	248 (55)	
Number of positive cores			**0.03**
One	232 (26)	83 (18)	
Two	163 (18)	96 (21)	
Three	135 (15)	70 (15)	
Four or more	202 (23)	114 (27)	
Biopsy Gleason sum			**<0.001**
3 + 3	570 (64)	335 (74)	
3 + 4	325 (36)	118 (26)	

*P-value from chi-square test.

**Table 3:** Multivariate logistic regression for risk of Gleason upgrading at radical prostatectomy.

	Odds ratio*	95% CI
Site of biopsy		
Academic	1.00	Referent
Community	0.91	0.68–1.22 (*P* = 0.53)
Age at biopsy		
<60	1.00	Referent
60–69	1.38	**1.06**–**1.82 (*P* = 0.02)**
≥70	2.82	**1.87**–**4.28 (*P* < 0.01)**
PSA		
<4.0	1.00	Referent
4–9.9	1.77	**1.27**–**2.47 (*P* < 0.01)**
≥10	4.08	**2.11–6.35 (*P* < 0.01)**
Prostate size (cc)		
<35	1.00	Referent
35–44	0.80	0.56–1.12 (*P* = 0.2)
44–56	0.65	**0.45–0.93 (*P* = 0.02)**
>56	0.51	**0.35–0.74 (*P* = 0.04)**
Total number of biopsy cores		
<10	1.00	Referent
≥10	0.85	0.61–1.19 (*P* = 0.5)
Number of positive cores		
One	1.00	Referent
Two	1.70	**1.15–2.50 (*P* = 0.01)**
Three	1.53	**1.00–2.34 (*P* = 0.04)**
Four or more	1.96	**1.33–2.89 (*P* < 0.01)**
Biopsy Gleason sum		
3 + 3	1.00	Referent
3 + 4	0.39	**0.29–0.52 (*P* < 0.01)**

*Odds ratios adjusted for all other variables in the table.
